# Insurance status and cancer treatment mediate the association between race/ethnicity and cervical cancer survival

**DOI:** 10.1371/journal.pone.0193047

**Published:** 2018-02-15

**Authors:** Sarah C. Markt, Tianyu Tang, Angel M. Cronin, Ingrid T. Katz, Brooke E. Howitt, Neil S. Horowitz, Larissa J. Lee, Alexi A. Wright

**Affiliations:** 1 Department of Epidemiology, Harvard T.H. Chan School of Public Health, Boston, Massachusetts, United States of America; 2 Dana-Farber Cancer Institute, Boston, Massachusetts, United States of America; 3 Harvard Medical School, Boston, Massachusetts, United States of America; 4 Department of Medicine, Brigham and Women’s Hospital, Boston, Massachusetts, United States of America; 5 Center for Global Health, Massachusetts General Hospital, Boston, Massachusetts, United States of America; 6 Department of Pathology, Brigham and Women’s Hospital, Boston, Massachusetts, United States of America; 7 Department of Obstetrics and Gynecology, Division Gynecologic Oncology, Brigham and Women’s Hospital, Boston, Massachusetts, United States of America; 8 Department of Radiation Oncology, Brigham and Women’s Hospital, Boston, Massachusetts, United States of America; Rudjer Boskovic Institute, CROATIA

## Abstract

Cervical cancer outcomes remain poor among disadvantaged populations, including ethnic minorities, low-income, and underinsured women. The aim of this study was to evaluate the mechanisms that underlie the observed association between race/ethnicity and cervical cancer survival. We identified 13,698 women, ages 21 to 64 years, diagnosed with stages I-III primary cervical cancer between 2007–2013 in Surveillance, Epidemiology, and End Results (SEER). Multivariable Cox proportional hazards regression models evaluated associations between race/ethnicity (Non-Hispanic White, Non-Hispanic Black, Hispanic, Other) and cervical cancer-specific mortality. We conducted mediation analysis to calculate the mediation proportion and its 95% confidence interval. Non-Hispanic black women had an increased risk of cervical cancer-specific mortality (HR: 1.23, 95% CI: 1.08–1.39), and Hispanic women a decreased risk of dying from their disease (HR: 0.82, 95% CI: 0.72–0.93), compared with non-Hispanic white. The estimated proportion of excess cervical cancer mortality for non-Hispanic black women relative to non-Hispanic white women that was mediated by insurance was 18.6% and by treatment was 47.2%. Furthermore, non-Hispanic black women were more likely to receive radiation and less likely to receive surgery for early-stage disease. In this population-based study we found that some of the excess cervical cancer-specific mortality for non-Hispanic black women is mediated by factors such as insurance status and treatment. These findings suggest that enhancing existing insurance coverage and ensuring equal and adequate treatment in all women may be a key strategy for improving cervical cancer outcomes.

## Introduction

Cervical cancer is the third most prevalent cancer among women and the fourth most common cause of cancer-related deaths worldwide[1, 2]. In the United States, the incidence of cervical cancer has decreased dramatically due to the widespread uptake of the Papanicolaou smear in routine screening and treatment of high-grade precursor lesions[3]. Despite this, the incidence of cervical cancer remain substantial, particularly among black and Hispanic women and in populations in Southern regions[3–5]. Women who are uninsured or do not have a regular health care provider remain at higher risk for developing this disease[3, 6, 7]. To address these disparities, a number of federal and state programs have aimed to increase cervical cancer screening rates and improve access to care for disadvantaged populations, including ethnic minorities, low-income, underinsured, and uninsured women.

Churilla *et al* [7]. recently demonstrated that cervical cancer patients with Medicaid insurance and those without insurance had increased odds of having advanced stage disease at diagnosis, receiving suboptimal therapy, and experiencing an increased risk of overall mortality, compared to those with private insurance[7]. Churilla *et al*. also found that black women were more likely to have Medicaid or lack of insurance, compared with white women[7], but did not specifically test the interaction of race and insurance status. Prior research has shown that black women are more likely to be diagnosed with advanced-stage cervical cancer and are less likely to receive timely and adequate cervical cancer treatment[8–13]. A recent study in the Surveillance, Epidemiology and End Results (SEER) data showed that among women with cervical cancer, black women were more likely to die from their disease compared to white women, and adjustment for sociodemographic and clinical characteristics attenuated the results[14]. To date, however, researchers have not examined the proportion of cervical cancer mortality in non-Hispanic black women and Hispanic women relative to non-Hispanic white women that can be attributed to intermediate variables, such as insurance status.

The aim of this study was to evaluate the mechanisms that underlie the observed association between race/ethnicity and cervical cancer survival. In doing so, we aimed to examine the mediation of the association between race/ethnicity and cervical cancer survival by modifiable clinical and socioeconomic factors, with a particular interest in insurance status, within the SEER database.

## Methods

### Data source and study population

The SEER18 registries data cover approximately 28% of the United States population, and collect information on patient demographics, tumor characteristics, treatment and survival for all incident cases[15]. The population covered is comparable to the general U.S. population.

We included all cases of invasive cervical cancer diagnosed between January 1, 2007 and December 31, 2013 (N = 23,891). We excluded cases that were diagnosed at autopsy or death (N = 133), those missing information on race (N = 135), and those with missing information on insurance status (N = 1,033), <21 years of age (and therefore ineligible for cervical cancer screening) or ≥65 years of age [since all were Medicare-eligible, but may have had other insurance (N = 4,389)]. We excluded older patients because we were primarily interested in disparities in younger women. We also excluded patients diagnosed with multiple cancers (N = 1,875), with a histologic subtype suggestive of another primary site (N = 585) or with implausible values for survival time (N = 40). Finally, we excluded women diagnosed with stage IV disease (N = 2,003) because of lack of chemotherapy treatment data in SEER. The final analytic cohort included 13,698 women.

### Outcome

The primary outcome of interest was cervical cancer-specific mortality. All-cause and cervical cancer-specific mortality were extracted from SEER cause-specific death and other cause of death classification. Survival time was measured in months from the date of diagnosis to death from any cause or cervical cancer, or to the end of follow-up.

### Exposure classification

The primary exposure of interest was race/ethnicity categorized as non-Hispanic white, non-Hispanic black, Hispanic or other (American Indian/AK Native, Asian/Pacific Islander).

### Mediators

The primary mediator of interest was insurance status, which was defined as uninsured or Medicaid insurance (i.e. any Medicaid or Indian/public health service), or private insurance (i.e. fee-for-service, managed care, health maintenance organization, preferred provider organization, TRICARE, and Medicare). We hypothesized that insurance status would be the driver of racial disparities in cervical cancer mortality, independent of other covariates. However, we also evaluated other potential mediators such as: marital status [married vs. unmarried (single, divorced, widowed)], region (West, Northeast, South, Midwest), and county-level measures of income (median household income; quartiles) and education (% high school completion; quartiles), obtained from linked Census 2008–12 American Community Survey data.

Clinically important potential mediators included stage at diagnosis and treatment. Cancer stage at diagnosis was defined based on the American Joint Committee on Cancer staging atlas (6^th^ ed.), and categorized into stages I, II, III. Treatment information was limited to surgery and/or radiation therapy (XRT), as SEER does not release chemotherapy information. Receipt of surgery was defined based on SEER site-specific surgery of primary site codes. XRT was defined as beam radiation, radioactive implants, radioisotopes, radiation with method not specified, or a combination of beam with implants or isotopes. We categorized treatment as surgery only, radiation only, both surgery and radiation, and neither treatment.

### Statistical analysis

We examined the association between race and baseline characteristics using a Pearson chi-square test. We conducted Cox proportional hazards models to estimate the adjusted association between race and death from any cause and cervical cancer-specific death. The multivariable model was adjusted for all baseline characteristics, stage at diagnosis and treatment received.

We conducted a mediation analysis to calculate the mediation proportion and its 95% confidence interval[16, 17]. The mediation proportion is the proportion of excess risk of cervical cancer mortality in non-Hispanic black women relative to non-Hispanic white women that can be attributed to the intermediate variable (e.g. lack of insurance) in black women. Similarly, it is the proportion of reduced risk of cervical cancer mortality in Hispanic women relative to non-Hispanic white women that can be attributed to the intermediate variable. We also conducted sensitivity analyses using other methods and our results were similar[18, 19]. Each mediation model is adjusted for the other mediators evaluated (i.e. the mediation model for insurance status is adjusted for marital status, education, region, income, stage and treatment), and age and year at diagnosis.

We then evaluated differences in treatment for early and late stage disease by race/ethnicity. For this analysis, we categorized stage as: (1) Early stage disease: stages 1A, 1A1, 1A2, 1B, 1B1, 1B2, 1NOS, and (2) Late stage disease: stages 2A, 2B, 2NOS, 3A, 3B, 3NOS.

Finally, we conducted multivariable Cox proportional hazards regression models to calculate hazard ratios (HRs) and 95% confidence intervals (95% CIs) for the association between region and cervical cancer mortality, separately for Hispanic and non-Hispanic black women. The multivariable models were adjusted for age at diagnosis, insurance status, marital status, education, income, region, year of diagnosis, histology, grade, stage and treatment.

Two-sided p-values <0.05 were considered statistically significant. All statistical analyses were performed with SAS software, version 9.4 (SAS Institute, Cary, NC).

## Results

Among the 13,698 women diagnosed with stage I-III cervical cancer between 2007 and 2013, 7,234 (53%) self-reported as non-Hispanic white, 1,823 (13%) as non-Hispanic black, 3,323 (24%) as Hispanic and 1,318 (10%) as other race/ethnicity. A higher percentage of Non-Hispanic black women had Medicaid insurance or no insurance, were unmarried, and lived in the South and in counties with lower median household income, compared with non-Hispanic white women (Table 1). Fewer non-Hispanic black women were diagnosed with a stage I tumor and more had squamous cell carcinoma, compared with non-Hispanic white women (Table 1). Compared to non-Hispanic white women, a higher percentage of Hispanic women had Medicaid insurance or no insurance, lived in the West, and had lower education levels (Table 1). Stage, grade and histology were similar between non-Hispanic white, Hispanic, and other race women.

**Table 1:** Sociodemographic and clinical characteristics by race of women with cervical cancer in SEER, 2007–2013.

Characteristics	Non-Hispanic White	Non-Hispanic Black	Hispanic	Other
	(n = 7,234)	(n = 1,823)	(n = 3,323)	(n = 1,318)
**Insurance**				
Private	5,133 (71%)	900 (49%)	1,397 (42%)	839 (64%)
Medicaid	1,631 (23%)	705 (39%)	1,500 (45%)	405 (31%)
Uninsured	470 (7%)	218 (12%)	426 (13%)	74 (6%)
**Age**				
21–34	1,417 (20%)	318 (17%)	767 (23%)	185 (14%)
35–44	2,451 (34%)	557 (31%)	1,182 (36%)	410 (31%)
45–54	2,017 (28%)	561 (31%)	876 (26%)	411 (31%)
55–64	1,349 (19%)	387 (21%)	498 (15%)	312 (24%)
**Year of Diagnosis**				
2007	1,093 (15%)	268 (15%)	486 (15%)	196 (15%)
2008	1,063 (15%)	274 (15%)	495 (15%)	175 (13%)
2009	1,083 (15%)	267 (15%)	489 (15%)	172 (13%)
2010	1,043 (14%)	253 (14%)	473 (14%)	193 (15%)
2011	1,027 (14%)	249 (14%)	468 (14%)	178 (14%)
2012	969 (13%)	261 (14%)	468 (14%)	198 (15%)
2013	956 (13%)	251 (14%)	444 (13%)	206 (16%)
**Marital Status**				
Unmarried	3,612 (50%)	1,388 (76%)	1,871 (56%)	552 (42%)
Married	3,622 (50%)	435 (24%)	1,452 (44%)	766 (58%)
**Geographic Region**				
West	3,205 (44%)	407 (22%)	2,707 (81%)	1,139 (86%)
Northeast	1,100 (15%)	319 (18%)	397 (12%)	97 (7%)
South	2,217 (31%)	871 (48%)	166 (5%)	58 (4%)
Midwest	712 (10%)	226 (12%)	53 (2%)	24 (2%)
**Residence**				
Metropolitan	6,109 (85%)	1,667 (91%)	3,228 (97%)	1,224 (93%)
**High School Completion (%)**^1^				
Quartile 1 (<78%)	1,377 (19%)	357 (20%)	1,353 (41%)	318 (24%)
Quartile 2 (78%-85%)	1,665 (23%)	682 (37%)	876 (26%)	204 (15%)
Quartile 3 (86%-88%)	1,802 (25%)	492 (27%)	681 (20%)	373 (28%)
Quartile 4 (>88%)	2,390 (33%)	292 (16%)	413 (12%)	423 (32%)
**Median Household Income ($)**^1^				
Quartile 1 (<$59,530)	2,101 (29%)	704 (39%)	501 (15%)	100 (8%)
Quartile 2 ($59530-$64019)	1,267 (18%)	438 (24%)	1,336 (40%)	346 (26%)
Quartile 3 ($64020-$79960)	2,029 (28%)	409 (22%)	728 (22%)	294 (22%)
Quartile 4 (>$79960)	1,837 (25%)	272 (15%)	758 (23%)	578 (44%)
**Stage at Diagnosis**				
I	4,332 (60%)	844 (46%)	1,869 (56%)	723 (55%)
II	912 (13%)	326 (18%)	427 (13%)	198 (15%)
III	1,582 (22%)	502 (28%)	786 (24%)	328 (25%)
Unknown	408 (5%)	151 (8%)	241 (7%)	69 (5%)
**Histology**				
Squamous cell carcinoma	4,594 (64%)	1,463 (80%)	2,251 (68%)	836 (63%)
Adenocarcinoma	1,357 (19%)	126 (7%)	546 (16%)	242 (19%)
Other	1,283 (18%)	234 (13%)	526 (16%)	240 (18%)
**Tumor Grade**				
I: Well differentiated	953 (13%)	132 (7%)	362 (11%)	164 (12%)
II: Moderately differentiated	2,369 (33%)	576 (32%)	1,072 (32%)	397 (30%)
III-IV: Poorly differentiated or undifferentiated	1,914 (26%)	555 (30%)	974 (29%)	375 (28%)
Unknown	1,998 (28%)	560 (31%)	915 (28%)	382 (29%)

^1^County-level data: percent of population with high school completion and median household income

### Cervical cancer-specific and all-cause mortality

Of the women diagnosed with potentially curable cervical cancer (stages I-III), 2,208 (16%) died between 2007 and 2013, with the majority dying from cervical cancer (n = 1,906, 86%). The median survival time was 31 months.

Women who were non-Hispanic black had a statistically significant increased risk of all-cause and cervical cancer-specific mortality, compared with non-Hispanic white women (All-cause HR: 1.23, 95% CI: 1.09–1.38; cervical cancer-specific mortality HR: 1.23, 95% CI: 1.08–1.39) (Table 2). Hispanic women had a 20% reduced risk of all-cause and cervical cancer-specific mortality (Table 2), compared with non-Hispanic white women. While other race women also had a reduced risk of mortality, the association was not statistically significant (Table 2).

**Table 2:** Association between race and all-cause and cervical cancer mortality, among women diagnosed with stage I-III disease in SEER 2007–2013.

	Non-Hispanic White	Non-Hispanic Black	Hispanic	Other
**All-cause Mortality**				
N cases	1143	453	441	171
HR (95% CI)*	Ref.	1.23 (1.09–1.38)	0.80 (0.71–0.91)	0.85 (0.72–1.01)
**Cervical Cancer Mortality**				
N cases	975	381	397	153
HR (95% CI)*	Ref.	1.23 (1.08–1.39)	0.82 (0.72–0.93)	0.88 (0.74–1.05)

*Adjusted for insurance status, stage, treatment, age, year of diagnosis, marital status, tumor grade, geographic region, urban/rural residence, histology, education and income.

### Mediation

There was evidence of statistically significant mediation of the racial disparities in cervical cancer mortality by modifiable socioeconomic and clinical variables. The estimated proportion of excess cervical cancer mortality for non-Hispanic black women relative to non-Hispanic white women that was mediated by socioeconomic variables was 18.6% for insurance, 14.6% for marital status, 6.2% for education, and 8.1% for region (Table 3); income was a non-statistically significant mediator (2.7%; p = 0.08). Furthermore, the proportion mediated by stage was 22.0% and by treatment was 47.2% (Table 3).

**Table 3:** Proportion mediated^1^ of the association between race and cervical cancer mortality by socioeconomic and clinical variables, among women diagnosed with stages I-III disease in SEER 2007–2013*.

	Non-Hispanic White	Non-Hispanic Black	Hispanic
Insurance	Ref.	18.6% (11.8%-28.2%)	-----^2^
		p <0.001	
Marital Status	Ref.	14.6% (8.7%-23.5%)	2.0% (0.0%-72.0%)
		p <0.001	p = 0.34
Education	Ref.	6.2% (2.7%-13.8%)	-----^2^
		p = 0.004	
Income	Ref.	2.7% (0.6%-11.4%)	-----^2^
		p = 0.08	
Region	Ref.	8.1% (3.0%-20.0%)	23.8% (10.3%-46.0%)
		p = 0.01	p <0.001
Stage at diagnosis (Stages I, II, III)	Ref.	22.0% (8.4%-46.5%)	10.3% (0.6%-69.1%)
		p = 0.01	p = 0.23
Treatment	Ref.	47.2% (27.9%-67.4%)	-----^2^
		p <0.001	

*Other race removed because none of the factors were mediators

^1^ All mediation models are mutually adjusted for the other potential mediators (i.e. the model for mediation by insurance status is adjusted for marital status, education, income, region, stage and treatment), and age and year at diagnosis.

^2^ ----- = Not mediated

Among Hispanic women, we did not find evidence of mediation by insurance status. The proportion of reduced cervical cancer mortality for Hispanic women mediated by region was surprisingly strong (23.8%) (Table 3). As shown in S1 Table, among Hispanic women, those living in the northeast had a statistically significant increased risk of cervical cancer-specific mortality, compared with those living in the West, while those living in the South and Midwest had a non-statistically significant reduced risk of cervical cancer-specific mortality. Among non-Hispanic black women, there was no association between region and cervical cancer-specific mortality (S1 Table).

### Treatment differences by race

We did not expect to see such a strong mediation effect by treatment among non-Hispanic black women, and therefore wanted to evaluate potential differences in receipt of treatment by race. Among women diagnosed with early stage disease, non-Hispanic black women were more likely to receive radiation and less likely to receive surgery, which is the standard of care (Table 4). Similarly, among late stage disease, non-Hispanic black women were less likely to receive surgery (Table 4). The percentages receiving radiation across races were similar for late stage disease. In both late and early stage disease, the proportion of Hispanic women receiving radiation and surgery was similar to non-Hispanic white women (Table 4).

**Table 4:** Treatment given for cervical cancer by race, stratified by stage of disease at diagnosis.

**Early Stage Disease (Stage 1A, 1A1, 1A2, 1B, 1B1, 1B2, 1NOS)**				
	**Non-Hispanic White**	**Non-Hispanic** **Black**	**Hispanic**	**Other**
	**(n = 4,332)**	**(n = 844)**	**(n = 1,869)**	**(n = 723)**
**Radiation**				
EBRT + Brachytherapy	482 (11%)	109 (13%)	167 (9%)	59 (8%)
EBRT alone	468 (11%)	128 (15%)	238 (13%)	86 (12%)
Brachy alone	75 (2%)	41 (5%)	47 (3%)	14 (2%)
Radiation NOS	3 (0.1%)	3 (0.4%)	10 (0.5%)	1 (0.1%)
No radiation	3,228 (75%)	543 (64%)	1,388 (74%)	552 (76%)
Unknown/refused	76 (2%)	20 (2%)	19 (1%)	11 (2%)
**Surgical Management**				
No surgery	378 (9%)	142 (17%)	231 (12%)	57 (8%)
Radical hysterectomy	715 (17%)	96 (11%)	294 (16%)	144 (20%)
Local destruction	726 (17%)	154 (18%)	281 (15%)	125 (17%)
TAH	592 (14%)	95 (11%)	276 (15%)	75 (10%)
TAH-BSO	943 (22%)	199 (24%)	417 (22%)	164 (23%)
Modified radical and/or extended hysterectomy	714 (16%)	105 (12%)	288 (15%)	121 (17%)
Hysterectomy NOS	235 (5%)	48 (6%)	75 (4%)	34 (5%)
Pelvic exenteration	4 (0.1%)	0 (0%)	1 (0.1%)	1 (0.1%)
Surgery NOS	9 (0.2%)	1 (0.1%)	1 (0.1%)	1 (0.1%)
Surgery Unknown	5 (0.2%)	1 (0.1%)	0 (0%)	0 (0%)
Other	11 (0.3)	3 (0.4%)	5 (0.3%)	1 (0.1%)
**Late Stage Disease (Stage 2A, 2B, 2NOS, 3A, 3B, 3NOS)**				
	**Non-Hispanic White**	**Non-Hispanic** **Black**	**Hispanic**	**Other**
	**(n = 2,494)**	**(n = 828)**	**(n = 1,213)**	**(n = 526)**
**Radiation**				
EBRT + Brachytherapy	1,152 (46%)	304 (37%)	487 (40%)	228 (43%)
EBRT alone	912 (37%)	337 (41%)	489 (40%)	191 (36%)
Brachy alone	200 (8%)	83 (10%)	111 (9%)	44 (8%)
Radiation NOS	13 (0.5%)	8 (1%)	11 (1%)	5 (1%)
No radiation	170 (7%)	70 (8%)	95 (8%)	48 (9%)
Unknown/refused	47 (2%)	26 (3%)	20 (2%)	10 (2%)
**Surgical Management**				
No surgery	1,575 (63%)	621 (75%)	798 (66%)	331 (63%)
Radical hysterectomy	199 (8%)	25 (3%)	101 (8%)	54 (10%)
Local destruction	213 (9%)	85 (10%)	92 (8%)	35 (7%)
TAH	33 (1%)	10 (1%)	29 (2%)	6 (1%)
TAH-BSO	206 (8%)	37 (4%)	85 (7%)	54 (11%)
Modified radical and/or extended hysterectomy	176 (7%)	29 (4%)	67 (6%)	30 (6%)
Hysterectomy NOS	60 (2%)	15 (2%)	27 (2%)	10 (2%)
Pelvic exerteration	10 (0.4%)	1 (0.1%)	3 (0.3%)	1 (0.2%)
Surgery NOS	14 (0.6%)	3 (0.4%)	10 (0.8%)	5 (1%)
Surgery Unknown	5 (0.2%)	2 (0.2%)	0 (0%)	0 (0%)
Other	3 (0.1%)	0 (0%)	1 (0.1%)	0 (0%)

## Discussion

In this study we found that, similar to previously published literature[9, 10, 14, 20], non-Hispanic black women were more likely to die of cervical cancer, compared with non-Hispanic white women, and Hispanic women had a reduced risk of cervical cancer-mortality, relative to non-Hispanic white women. Furthermore, we found that several potentially modifiable factors, including insurance status and treatment, were important mediators of the association between race/ethnicity and cervical cancer mortality.

Many individual, socioeconomic, and healthcare provider factors have previously been associated with worse cervical cancer-specific survival in older women[20]. Our study extends these results by demonstrating that these factors are also important in younger women, the population at the highest risk for developing cervical cancer. Our finding that the estimated proportion of excess cervical cancer mortality for non-Hispanic black women relative to non-Hispanic white women is mediated by potentially modifiable factors, such as insurance status and treatment, further extends earlier research investigating potential mechanisms underlying racial disparities in cervical cancer survival.

Earlier work has focused on examining associations of race, ethnicity and socioeconomic status with disparities in the stage of disease at diagnosis, treatment, and cervical cancer-specific survival[8, 21–24]. Here, we show that after adjusting for important socioeconomic and clinical factors, the excess cervical cancer mortality in non-Hispanic black women compared with white women that can be attributed to insurance status was 18.6% among women diagnosed with stages I-III. This is important because insurance status is a characteristic that can be modified, as demonstrated recently by Robbins, et al. who found that implementation of the Affordable Care Act (ACA) Dependent Coverage Expansion in September 2010 was associated with a net increase in diagnosis of early-stage disease and use of fertility-sparing treatments among women aged 21 to 25 years, but not among women aged 26–34, compared with earlier years[23]. Furthermore, the expansion of insurance coverage, including coverage of preventative services such as the HPV vaccine, may also help mitigate some of the adverse outcomes. These findings suggest that enhanced insurance coverage may mitigate existing inferior cervical cancer outcomes associated with non-private health insurance. Conversely, efforts to limit health insurance coverage may exacerbate disparities in outcomes.

We also showed a large proportion of the association between race/ethnicity and cervical cancer mortality was mediated by treatment and region. Previous studies have found conflicting results for potential racial disparities in treatment. Some studies have shown black women receive less treatment[10, 11, 13, 25–28], even after adjusting for age, stage, histology and SES, while others have found no association in treatment according to race[29, 30]. A randomized trial found no differences in overall or disease-free survival comparing radiation and surgery in women with stages Ib-IIa cervical cancer[31]. However, full adherence to the radiation regime was attained in the setting of a randomized trial, which may not reflect clinical practice. Observational studies suggest potential differences in adherence to the full treatment course. Furthermore, a recent study by Beavis *et al*. showed cervical cancer mortality rates were underestimated after correcting for hysterectomy, particularly among black women[32]. Here, we show that a large proportion (47%) of the excess cervical cancer mortality in black women can be explained by treatment, and that black women are more likely to receive radiation and less likely to receive surgery for early stage disease.

Region was also found to be a potential mediator of the association between racial disparities and cervical cancer mortality, for both non-Hispanic and Hispanic women. Sheppard *et al*. showed differences in the association between race and survival between states[30]. Similarly, distance from Comprehensive Cancer Centers has previously been shown to be associated with poor outcomes, including a decreased likelihood of completing a full treatment regime and increased risk of death[33]. In this study, region and treatment mediated the association between race and cervical cancer-specific survival independently, suggesting that both are potential targets for improved outcomes. The association with region could also be the result of chance given fewer deaths from cervical cancer in regions other than the West. Alternatively, our results may be related to differences in acculturation by geographic region. Previous studies have shown that differences in language, culture, and low health literacy are barriers adequate healthcare, including accessing fewer preventative services and lack of adherence to treatment regimes.[34–37]

There are a few limitations to this study worth noting. First, SEER does not release information on chemotherapy, which limited our ability to ascertain whether patients with stages I-III disease received concurrent chemotherapy with radiation, or whether these associations remained true for patients with stage IV disease. However, data from previous studies suggest that there has been rapid and widespread adoption of concurrent chemotherapy and radiation since 1999 when three randomized clinical trials demonstrated improved survival compared with radiation alone[38, 39]. Second, our study population was restricted to women aged 21 to 64 years so our results may not apply to younger or older patients; however, this age range captures approximately 80% of all cervical cancer cases diagnosed in the U.S.[40] Finally, we note that race/ethnicity and socioeconomic factors are complex constructs with both individual and group-level determinants. We attempted to evaluate and adjust for factors thought to be important potential pathways in racial disparities in cervical cancer mortality; however, we were limited by county-level indices of income and education and lack of detailed information about treatment decision-making, including patient preferences.

In closing, our study suggests that some of the excess mortality for non-Hispanic black women and reduced mortality for Hispanic women, relative to non-Hispanic white women, is mediated by factors such as insurance status, region and treatment. These findings suggest that enhancing existing insurance coverage and ensuring equal and adequate treatment in all women may be a key strategy for improving cervical cancer outcomes among non-elderly women. In addition, continued expansion of preventative services, such as immunization against HPV, a known cause of cervical cancer, should be encouraged.

## Supporting information

S1 Table Association between region and cervical cancer mortality, among Hispanic women and non-Hispanic Black women (stages I-III). *Multivariable model adjusted for age at diagnosis, insurance status, marital status, education, income, region, year of diagnosis, histology, grade, stage and treatment.
(DOCX)

## References

[1] Ferlay J, Soerjomataram I, Dikshit R, Eser S, Mathers C, Rebelo M, et al Cancer incidence and mortality worldwide: sources, methods and major patterns in GLOBOCAN 2012. Int J Cancer. 2015;136(5):E359–86. doi: 10.1002/ijc.29210 . 25220842 10.1002/ijc.29210

[2] Bray F, Ren JS, Masuyer E, Ferlay J. Global estimates of cancer prevalence for 27 sites in the adult population in 2008. Int J Cancer. 2013;132(5):1133–45. doi: 10.1002/ijc.27711 . 22752881 10.1002/ijc.27711

[3] (CDC) CfDCaP. Vital signs: cervical cancer incidence, morality and screening—United States, 2007–2012. 2014. PMC5779486 25375072

[4] Cervical Cancer Rates by Race and Ethnicity. In: Control CfD, editor. 2015.

[5] Ward E, Jemal A, Cokkinides V, Singh GK, Cardinez C, Ghafoor A, et al Cancer disparities by race/ethnicity and socioeconomic status. CA Cancer J Clin. 2004;54(2):78–93. . 15061598 10.3322/canjclin.54.2.78

[6] Cowburn SC, M.J.; Lapidus J.A.; DeVoe J.E. The Association Between Insurance Status and Cervical Cancer Screening in Community Health Centers: Exploring the Potential of Electronic Health Records for Population-Level Surveillance, 2008–2010. Prev Chronic Dis. 2013;10. 10.5888/pcd10.130034 PMC3809921 24157076

[7] Churilla T, Egleston B, Dong Y, Shaikh T, Murphy C, Mantia-Smaldone G, et al. Disparities in the management and outcome of cervical cancer in the United States according to health insurance status. (1095–6859 (Electronic)). doi: D—NLM: NIHMS772744 [Available on 06/01/17] D—NLM: PMC4877265 [Available on 06/01/17] EDAT- 2016/03/26 06:00 MHDA- 2016/03/26 06:00 CRDT- 2016/03/26 06:00 PMCR- 2017/06/01 PHST- 2015/12/08 [received] PHST- 2016/03/12 [revised] PHST- 2016/03/18 [accepted] AID - S0090-8258(16)30081-6 [pii] AID—10.1016/j.ygyno.2016.03.025 [doi] PST—ppublish. 10.1016/j.ygyno.2016.03.025 PMC4877265 27012428

[8] Simard EP, Fedewa S, Ma J, Siegel R, Jemal A. Widening socioeconomic disparities in cervical cancer mortality among women in 26 states, 1993–2007. Cancer. 2012;118(20):5110–6. doi: 10.1002/cncr.27606 22707306 10.1002/cncr.27606

[9] Eggleston KS, Coker AL, Williams M, Tortolero-Luna G, Martin JB, Tortolero SR. Cervical cancer survival by socioeconomic status, race/ethnicity, and place of residence in Texas, 1995–2001. J Womens Health (Larchmt). 2006;15(8):941–51. doi: 10.1089/jwh.2006.15.941 . 17087618 10.1089/jwh.2006.15.941

[10] Coker AL, Desimone CP, Eggleston KS, White AL, Williams M. Ethnic disparities in cervical cancer survival among Texas women. J Womens Health (Larchmt). 2009;18(10):1577–83. doi: 10.1089/jwh.2008.1342 ; PubMed Central PMCID: PMCPMC2825716. 19788363 10.1089/jwh.2008.1342 PMC2825716

[11] Howell EA, Chen YT, Concato J. Differences in cervical cancer mortality among black and white women. Obstet Gynecol. 1999;94(4):509–15. . 10511350 10.1016/s0029-7844(99)00334-8

[12] Rauh-Hain JA, Clemmer JT, Bradford LS, Clark RM, Growdon WB, Goodman A, et al Racial disparities in cervical cancer survival over time. Cancer. 2013;119(20):3644–52. doi: 10.1002/cncr.28261 . 23913530 10.1002/cncr.28261

[13] Patel DA, Barnholtz-Sloan JS, Patel MK, Malone JM Jr., Chuba PJ, Schwartz K. A population-based study of racial and ethnic differences in survival among women with invasive cervical cancer: analysis of Surveillance, Epidemiology, and End Results data. Gynecol Oncol. 2005;97(2):550–8. doi: 10.1016/j.ygyno.2005.01.045 . 15863159 10.1016/j.ygyno.2005.01.045

[14] Sheppard CS, El-Zein M, Ramanakumar AV, Ferenczy A, Franco EL. Assessment of mediators of racial disparities in cervical cancer survival in the United States. Int J Cancer. 2016;138(11):2622–30. doi: 10.1002/ijc.29996 . 26756569 10.1002/ijc.29996

[15] Surveillance, Epidemiology, and End Results (SEER) Program (http://www.seer.cancer.gov/) SEER*Stat Database: Incidence—SEER 18 Regs Research Data + Hurricane Katrine Impacted Louisianna Cases, Nov 2015 Sub (1973–2013) <Katrina/Rita Population Adjustment>—Linked To County Attributes—Total U.S., 1969–2012 Counties, National Cancer Institute, DCCPS, Surveillance Research Program, Surveillance Systems Branch, released April 2016, based on the November 2015 submission. [Internet].

[16] Lin DY, Fleming TR, De Gruttola V. Estimating the proportion of treatment effect explained by a surrogate marker. Stat Med. 1997;16(13):1515–27. . 9249922 10.1002/(sici)1097-0258(19970715)16:13<1515::aid-sim572>3.0.co;2-1

[17] Jun HJ, Austin SB, Wylie SA, Corliss HL, Jackson B, Spiegelman D, et al The mediating effect of childhood abuse in sexual orientation disparities in tobacco and alcohol use during adolescence: results from the Nurses' Health Study II. Cancer Causes Control. 2010;21(11):1817–28. doi: 10.1007/s10552-010-9609-3 ; PubMed Central PMCID: PMCPMC2962696. 20640883 10.1007/s10552-010-9609-3 PMC2962696

[18] Valeri L, VanderWeele TJ. SAS macro for causal mediation analysis with survival data. (1531–5487 (Electronic)). 10.1097/EDE.0000000000000253 25643116

[19] VanderWeele TJ. Causal mediation analysis with survival data. (1531–5487 (Electronic)). doi: D—NLM: NIHMS293732 D—NLM: PMC3109321 EDAT- 2011/06/07 06:00 MHDA- 2011/12/13 00:00 CRDT- 2011/06/07 06:00 AID—10.1097/EDE.0b013e31821db37e [doi] AID—00001648-201107000-00025 [pii] PST—ppublish. 10.1097/EDE.0b013e31821db37e PMC3109321 21642779

[20] Coker AL, Du XL, Fang S, Eggleston KS. Socioeconomic status and cervical cancer survival among older women: findings from the SEER-Medicare linked data cohorts. Gynecol Oncol. 2006;102(2):278–84. doi: 10.1016/j.ygyno.2005.12.016 16434087 10.1016/j.ygyno.2005.12.016

[21] Ward E, Halpern M, Schrag N, Cokkinides V, DeSantis C, Bandi P, et al Association of insurance with cancer care utilization and outcomes. CA: a cancer journal for clinicians. 2008;58(1):9–31. doi: 10.3322/CA.2007.0011 18096863 10.3322/CA.2007.0011

[22] Bradley CJ, Given CW, Roberts C. Health care disparities and cervical cancer. Am J Public Health. 2004;94(12):2098–103. 15569960 10.2105/ajph.94.12.2098 PMC1448598

[23] Robbins AS, Han X, Ward EM, Simard EP, Zheng Z, Jemal A. Association Between the Affordable Care Act Dependent Coverage Expansion and Cervical Cancer Stage and Treatment in Young Women. JAMA. 2015;314(20):2189–91. doi: 10.1001/jama.2015.10546 26599188 10.1001/jama.2015.10546

[24] Grant SR, Walker GV, Koshy M, Shaitelman SF, Klopp AH, Frank SJ, et al Impact of Insurance Status on Radiation Treatment Modality Selection Among Potential Candidates for Prostate, Breast, or Gynecologic Brachytherapy. Int J Radiat Oncol Biol Phys. 2015;93(5):968–75. doi: 10.1016/j.ijrobp.2015.08.036 . 26452570 10.1016/j.ijrobp.2015.08.036

[25] Mundt AJ, Connell PP, Campbell T, Hwang JH, Rotmensch J, Waggoner S. Race and clinical outcome in patients with carcinoma of the uterine cervix treated with radiation therapy. Gynecol Oncol. 1998;71(2):151–8. doi: 10.1006/gyno.1998.5203 . 9826453 10.1006/gyno.1998.5203

[26] Chatterjee S, Gupta D, Caputo TA, Holcomb K. Disparities in Gynecological Malignancies. Front Oncol. 2016;6:36 doi: 10.3389/fonc.2016.00036 ; PubMed Central PMCID: PMCPMC4761838. 26942126 10.3389/fonc.2016.00036 PMC4761838

[27] del Carmen MG, Montz FJ, Bristow RE, Bovicelli A, Cornelison T, Trimble E. Ethnic differences in patterns of care of stage 1A(1) and stage 1A(2) cervical cancer: a SEER database study. Gynecol Oncol. 1999;75(1):113–7. doi: 10.1006/gyno.1999.5543 . 10502436 10.1006/gyno.1999.5543

[28] Fleming S, Schluterman NH, Tracy JK, Temkin SM. Black and white women in Maryland receive different treatment for cervical cancer. PLoS ONE. 2014;9(8):e104344 doi: 10.1371/journal.pone.0104344 25121587 10.1371/journal.pone.0104344 PMC4133178

[29] Farley JH, Hines JF, Taylor RR, Carlson JW, Parker MF, Kost ER, et al Equal care ensures equal survival for African-American women with cervical carcinoma. Cancer. 2001;91(4):869–73. . 11241257

[30] Grigsby PW, Hall-Daniels L, Baker S, Perez CA. Comparison of clinical outcome in black and white women treated with radiotherapy for cervical carcinoma. Gynecol Oncol. 2000;79(3):357–61. doi: 10.1006/gyno.2000.5974 . 11104605 10.1006/gyno.2000.5974

[31] Landoni F, Maneo A, Colombo A, Placa F, Milani R, Perego P, et al Randomised study of radical surgery versus radiotherapy for stage Ib-IIa cervical cancer. Lancet. 1997;350(9077):535–40. doi: 10.1016/S0140-6736(97)02250-2 . 9284774 10.1016/S0140-6736(97)02250-2

[32] Beavis AL, Gravitt PE, Rositch AF. Hysterectomy-corrected cervical cancer mortality rates reveal a larger racial disparity in the United States. Cancer. 2017 doi: 10.1002/cncr.30507 . 28112816 10.1002/cncr.30507

[33] Barrington DA, Dilley SE, Landers EE, Thomas ED, Boone JD, Straughn JM Jr., et al Distance from a Comprehensive Cancer Center: A proxy for poor cervical cancer outcomes? Gynecol Oncol. 2016;143(3):617–21. doi: 10.1016/j.ygyno.2016.10.004 ; PubMed Central PMCID: PMCPMC5116397. 27720232 10.1016/j.ygyno.2016.10.004 PMC5116397

[34] Timmins CL. The impact of language barriers on the health care of Latinos in the United States: a review of the literature and guidelines for practice. J Midwifery Womens Health. 2002;47(2):80–96. . 12019990 10.1016/s1526-9523(02)00218-0

[35] Flores G. Language barriers to health care in the United States. N Engl J Med. 2006;355(3):229–31. doi: 10.1056/NEJMp058316 . 16855260 10.1056/NEJMp058316

[36] Schyve PM. Language differences as a barrier to quality and safety in health care: the Joint Commission perspective. J Gen Intern Med. 2007;22 Suppl 2:360–1. doi: 10.1007/s11606-007-0365-3 ; PubMed Central PMCID: PMCPMC2078554. 17957426 10.1007/s11606-007-0365-3 PMC2078554

[37] Echeverria SE, Carrasquillo O. The roles of citizenship status, acculturation, and health insurance in breast and cervical cancer screening among immigrant women. Med Care. 2006;44(8):788–92. doi: 10.1097/01.mlr.0000215863.24214.41 . 16862042 10.1097/01.mlr.0000215863.24214.41

[38] Pearcey R, Miao Q, Kong W, Zhang-Salomons J, Mackillop WJ. Impact of adoption of chemoradiotherapy on the outcome of cervical cancer in Ontario: results of a population-based cohort study. J Clin Oncol. 2007;25(17):2383–8. doi: 10.1200/JCO.2006.09.1926 . 17557951 10.1200/JCO.2006.09.1926

[39] Barbera L, Paszat L, Thomas G, Covens A, Fyles A, Elit L, et al The rapid uptake of concurrent chemotherapy for cervix cancer patients treated with curative radiation. Int J Radiat Oncol Biol Phys. 2006;64(5):1389–94. doi: 10.1016/j.ijrobp.2005.09.046 . 16413698 10.1016/j.ijrobp.2005.09.046

[40] SEER Cancer Statistics Factsheets: Cervix Uteri Cancer. National Cancer Institute. Bethesda, MD, http://seer.cancer.gov/statfacts/html/cervix.html [accessed May 17, 2016].

