# Supplementary material for: Insurance status and cancer treatment mediate the association between race/ethnicity and cervical cancer survival
Source: PLoS One. 2018 Feb 15;13(2):e0193047. doi: 10.1371/journal.pone.0193047 (PMC5814056; doi:10.1371/journal.pone.0193047)
Supplement: S1 Table — *Multivariable model adjusted for age at diagnosis, insurance status, marital status, education, income, region, year of diagnosis, histology, grade, stage and treatment. (DOCX) [file pone.0193047.s001.docx]

**Supplemental Table 1: Association between region and cervical cancer mortality, among Hispanic women and Non-Hispanic Black women (stages I-III)**

|  | **West** | **Northeast** | **South** | **Midwest** |
| --- | --- | --- | --- | --- |
| **Hispanics**  N Cases  HR (95% CI)* | 329  Ref. | 52  1.46 (1.06-2.02) | 11  0.80 (0.43-1.48) | 5  0.85 (0.34-2.11) |
| **Non-Hispanic Black**  N Cases  HR (95% CI)* | 88  Ref. | 86  1.20 (0.87-1.64) | 158  1.05 (0.72-1.53) | 49  1.48 (0.94-2.31) |

*Multivariable model adjusted for age at diagnosis, insurance status, marital status, education, income, region, year of diagnosis, histology, grade, stage and treatment.
